# Family Communication and Decision-Making in Feeding Options for Chinese American Persons With Advanced Dementia

**DOI:** 10.1093/geroni/igaf122.1125

**Published:** 2025-12-31

**Authors:** Jing Wang, Jing Huang, Bei Wu, Laura Hanson, Dena Schulman-Green, Yaolin Pei

**Affiliations:** University of New Hampshire, Durham, New Hampshire, United States; Johns Hopkins University, Baltimore, Maryland, United States; NYU Shanghai, Shanghai, China (People’s Republic); The University of North Carolina at Chapel Hill School of Medicine, Chapel Hill, North Carolina, United States; New York University, New York, New York, United States; The University of Texas at Austin, Austin, Texas, United States

## Abstract

Feeding difficulties commonly arise as dementia progresses to its advanced stages. Although research consistently shows that feeding tubes offer no clinical advantage, they continue to be widely used among Asian populations, particularly within the Chinese American community. This study employed a qualitative descriptive design to examine examines how family communication shaped feeding choices among 21 caregivers of Chinese American individuals with advanced dementia. Using semi-structured interviews, data were analyzed thematically to examine the influence of cultural values, family dynamics, and healthcare interactions in shaping feeding decisions. Findings highlight that feeding decisions are often made collectively, with strong deference to medical professionals and elder family members. Caregivers frequently struggle with navigating differing family perspectives, emotional distress, and decisional uncertainty, particularly in the absence of advance directives. Concerns about nutrition, prolonging life, and filial piety drive many families toward feeding tube insertion, even when caregivers express doubts. External pressures from healthcare providers and cultural expectations can further complicate decision-making. However, open family communication, shared values, and mutual understanding facilitated more cohesive decision-making, allowing families to align feeding choices with the perceived wishes of their loved ones. These findings underscore the critical need for culturally tailored decision-making support, early discussion of feeding preferences, and improved provider-family communication. Strengthening communication pathways within families and between families and healthcare providers can empower caregivers, reduce unnecessary feeding tube use, and enhance end-of-life care experiences for Chinese American families navigating advanced dementia care.

